# Adsorption of Heavy Metals Ions from Mining Metallurgical Tailings Leachate Using a Shell-Based Adsorbent: Characterization, Kinetics and Isotherm Studies

**DOI:** 10.3390/ma15155315

**Published:** 2022-08-02

**Authors:** Begoña Fernández Pérez, Julia Ayala Espina, María de Los Ángeles Fernández González

**Affiliations:** 1 Department of Materials Science and Metallurgical Engineering, University of Oviedo, 33004 Oviedo, Spain; jayala@uniovi.es; 2 Department of Geology, University of Oviedo, 33005 Oviedo, Spain; mafernandez@uniovi.es

**Keywords:** mollusks’ shell, heavy metal, adsorption, landfill leachate, wastewater treatment

## Abstract

This study defines the optimal parameters that allow the use of waste mollusk shells (WS) to remove heavy metals from three mining and metallurgical leachates. First, the influence of parameters such as pH, contact time, initial metal concentration, adsorbent dose and the presence of co-ions in Cu^2+^, Cd^2+^, Zn^2+^ and Ni^2+^ adsorption was investigated in synthetic solutions. Metal uptake was found to be dependent on the initial pH of the solution, the removal rate increasing with the increase in pH, showing the highest affinity at pH 5–6. The removal efficiency at lower concentrations was greater than at higher values. The competitive adsorption results on bimetallic solutions showed that the adsorption capacity of the sorbent was restricted by the presence of other ions and suppressed the uptake of heavy metals compared to the single adsorption. Cu^2+^ was the metal that most inhibited the removal of Cd^2+^, Zn^2+^ and Ni^2+^. The Langmuir isotherm provided the best fit to the experimental data for Cu^2+^, Cd^2+^ and Zn^2+^ and the Freundlich isotherm, for Ni^2+^. The data showed that the maximum adsorption capacity a_max_ for Zn^2+^, Cd^2+^ and Cu^2+^, was 526.32 mg g^−1^, 555.56 mg g^−1^ and 769.23 mg g^−1^, respectively. Sorption kinetics data best fit the pseudo-second-order kinetic model. The results obtained in the tests with three mining and metallurgical leachates showed that WS were effective in simultaneously removing several heavy metals ions such as Cu, Ni, Zn, Cd, Ni, As and Se.

## 1. Introduction

Asturias (northern Spain) is a region with a long-developed mining and metallurgical industry, which produces large quantities of contaminated water, including, for example, leachates from tailings dumps of abandoned, unrehabilitated facilities. These leachates and other leachates from operating metallurgical industries contain heavy metals that need to be treated. These elements are not biodegradable, they tend to accumulate in bottom sediments from which they may be released via diverse processes, and they can move up the biologic chain, thereby reaching human beings where they may produce genetic diseases or mutagenic or carcinogenic effects [1].

Conventional methods, including coagulation–flocculation, precipitation, filtration, liquid extraction, ion exchange, reverse osmosis, membrane separation and electrochemical treatment, have been used to remove or minimize the concentrations of heavy metal ions in industrial wastewater [2,3,4]. The adsorption method is considered the most efficient and economical due to its fast removal rate and minimum pretreatment of samples [5,6,7,8,9]. In recent years, various types of biocomposites have been used as biosorbents to remove heavy metals ions from solutions [10,11,12].

Several researchers have studied the removal of heavy metals with natural limestone [13,14,15]. In recent years, various biogenic calcium carbonate wastes have been used as low-cost adsorbents to remove heavy metals from solutions such as eggshells [16,17,18], oyster shells [19,20,21,22], crustacean shells [23], clam shells [24], *Anadara inaequivalvis* shells [25], golden apple snail shells [26] and cockle shells [27].

In 2019, 17,577,417 tons of aquaculture mollusks were harvested in the world. The European Union contributed 604,332 tons, or 3.4%, to this production. More than 7 million tons of mollusk shells are discarded each year as unwanted waste, and the vast majority of these shells are either thrown in landfills or dumped at sea [28]. The use of seashells as sorbents transforms a waste material into a by-product, making it a useful material, thus achieving a circular economy.

In order to develop low-cost and environmentally friendly technologies, the feasibility of the use of waste mollusk shells as a low-cost treatment for the removal of heavy metals ions from three mining and metallurgical waste leachates from different facilities located in northern Spain was investigated. This paper also presents the results of the removal of Cu^2+^, Zn^2+^, Cd^2+^ and Ni^2+^ from single and multicomponent solutions. The removal of heavy metal ions by this low-cost adsorbent was studied under various conditions.

## 2. Materials and Methods

### 2.1. Materials

The adsorbent used in this work was purchased from a Spanish company dedicated to the recycling of marine shells from the canning industry that was subjected to a continuous heat treatment of 135 °C for a period of 32 min in order to eliminate all harmful microorganisms. It consisted of crushed shells of different mollusks with a particle size of 4–0.5 mm. Prior to its use, a grinding process was carried out to obtain a homogeneous particle size. The waste mollusk shells (WS) were characterized by means of different instrumental technique: an X-ray diffraction analysis (PHILIPS X’ PERT PRO, Eindhoven, The Netherlands), a TGA thermogravimetric analysis (SDT Q 600, TA Instruments, New Castle, DE, USA) and a scanning electron microscope analysis (MEB JEOL-6610 LV, Akishima, Japan). The chemical composition was determined by mass spectrometry with inductively coupled plasma (ICP-MS Agilent 7700, Agilen, Santa Clara, CA, USA) prior to dissolution with aqua regia using an Microwave 3000 (Anton Paar, Graz, Austria) microwave system. The loss on ignition (LOI) was calculated by heating a preweighed dry sample to 900 °C for 3 h.

In the synthetic wastewater tests, all the chemical solutions employed were prepared using analytical grade metal sulfates and deionized water. Stock solutions were prepared containing 1000 mg L^−1^ Cu^2+^, Zn^2+^, Cd^2+^ and Ni^2+^, respectively. Solutions with the desired metal concentrations were prepared by successive dilutions of the stock solution. Before the experiment, the initial pH of aqueous solutions was adjusted by adding 1 M HNO_3_ and 1 M NaOH solutions.

The three mining and metallurgical leachates were analyzed by the inductively coupled plasma technique, in addition to determining their pH by using a PH2002 m (Crison^®^, pH-Meter BASIC20 CRISON, Barcelona, ES, USA). The sampling and analysis were in accordance with European standards (EN).

### 2.2. Batch Adsorption Experiments

Batch adsorption experiments were carried out by mechanically shaking series of 100 mL polyethylene bottles containing WS samples and metal solutions prepared in the laboratory using different adsorbent concentrations. The suspensions were shaken at room temperature (293 K) at 75 rpm, subsequently separating the two phases by filtration (Whatman 114 filter, Waltham, MA, USA). The solid residue was collected at the end of the reaction and dried.

The pH was measured and the concentrations of metal in the resulting supernatant were analyzed by atomic absorption spectroscopy (Perkin Elmer AAnalyst 200, Waltham, MA, USA). For each metal solution, one sample was reserved for analysis to determine the initial metal concentration. 

The amount of metal removed was determined by mass balances according to Equation (1):

(1) $$\%\;Metal_{removed}\;=\;\frac{\left(C_{o}\;-\;C_{e}\right)\;}{C_{o}}\times \;100$$

The amount of metal ion removed by WS (in milligrams per gram) was calculated according to Equation (2).

(2) $$q\;=\;\frac{\left(C_{o}^{\;}\;-\;C_{e}\right)\;\times V}{W_{s}}$$

where *q* is the amount of removed metal ion (mg g^−1^); *W_s_*, the amount of adsorbent (g); *C_o_* and *C_e_*, the metal ion concentration (mg L^−1^) before and after removal, respectively; and *V*, the sample volume (L).

Different series of batch experiments were carried out to determine the influence of pH, contact time, initial metal concentration, adsorbent dosage and the effect of other metal ions.

## 3. Results

### 3.1. Characterization of the Adsorbent

The WS are mainly composed of CaCO_3_ and various chlorides from seawater, 52.51% CaO, 43.37% LOI, 1.96% Cl, 1.01% Na, 0.20% K, 0.14% Sr, 0.04% Mg and 0.0024% SiO_2_.

**Figure 1:**
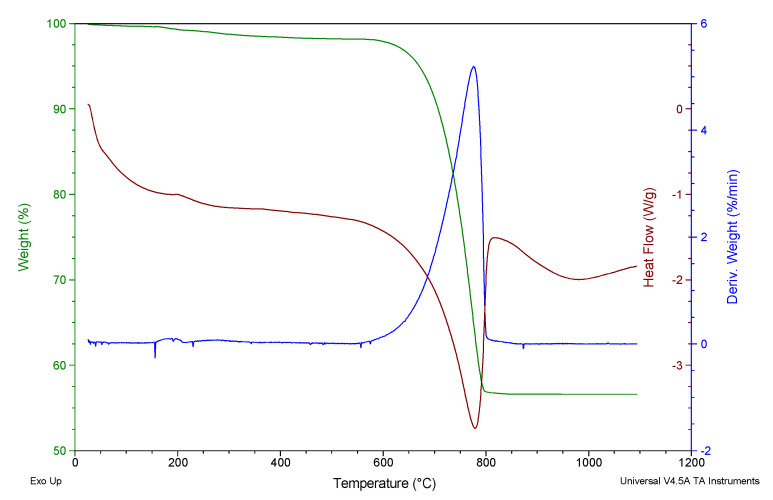
Thermogravimetric analysis of WS.

Figure 1 shows the results of the thermogravimetric analysis, a first phase up to approximately 500 °C where organic matter was lost (1.384 %wt). The organic matter was formed by proteins, glycoproteins and polysaccharides [29]. The next stage, between 600 and 800 °C corresponded to the decarbonation of calcium carbonate (41.56 %wt):

CaCO_3_(s)→CaO(s) + CO_2_(g) (3)

These values are similar to those found by other authors for this type of biogenic material [30]. These weight losses are in accordance with the LOI test.

**Figure 2:**
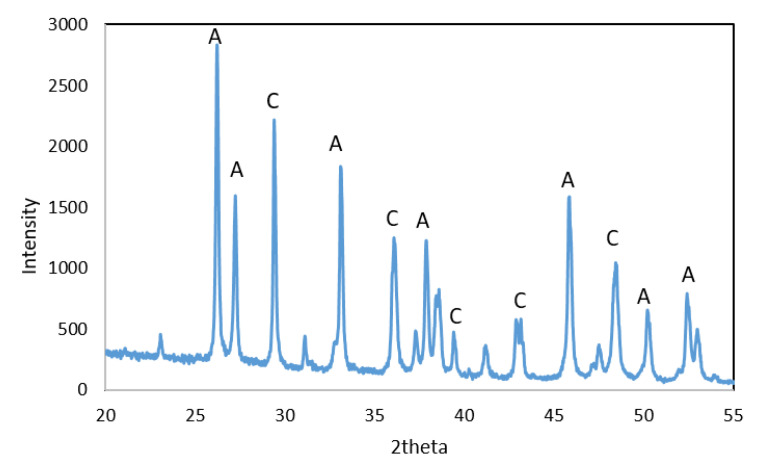
XRD diffractogram of WS: A—aragonite and C—calcite.

The X-ray diffraction analysis of WS shows that this residue consisted mainly of aragonite and calcite, Figure 2.

**Figure 3:**
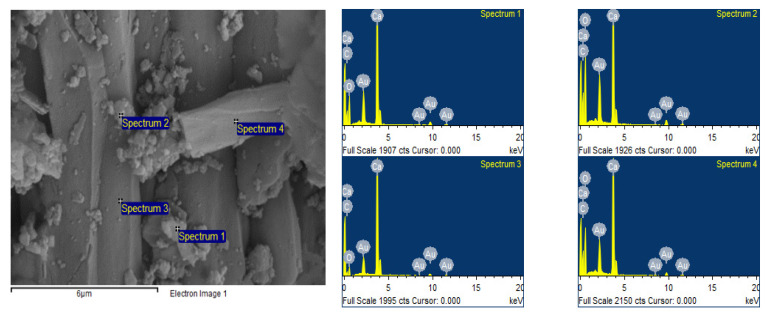
Scanning electron micrograph and EDAX spectrum of WS.

The SEM image shows that WS had large, elongated crystals with a smooth surface, along with small crystals which had a higher roughness. The EDAX spectra indicated that this adsorbent was primarily composed of C, O and Ca (Figure 3).

### 3.2. Characterization of Three Mining and Metallurgical Leachates

The three leachates were referenced as O, P and S. The leachates contained significant amounts of heavy metals such as Cd (67.61–16.69 mg L^−1^), Ni (20.82–8.25 mg L^−1^), Zn (22.29–15.11 mg L^−1^), Cu (27.66–0.020 mg L^−1^) and As (6.71–0.11 mg L^−1^), in addition to lower amounts of Pb, Se, Mn, Hg and Mo (66.64–0.46 µg L^−1^). They also had important amounts of alkaline ions (Na and K) and alkaline earth ions (Ca and Mg), Table 1. The pH values of the leachates O, P and S were also determined; the values were 5.4, 4.85 and 5.5, respectively.

### 3.3. Batch Adsorption Experiments

#### 3.3.1. Effect of pH

Initially, the effect of the initial pH solutions on the removal of metal ions was studied. For this purpose, a series of experiments were carried out using a solution concentration of 100 mg L^−1^ and different adsorbent concentration, 0.4, 4 and 10 g L^−1^. The samples were shaken at room temperature at 75 rpm for 24 h. The initial pH of the solutions was adjusted from 3 to 7 using H_2_SO_4_ and NaOH solutions.

**Figure 4:**
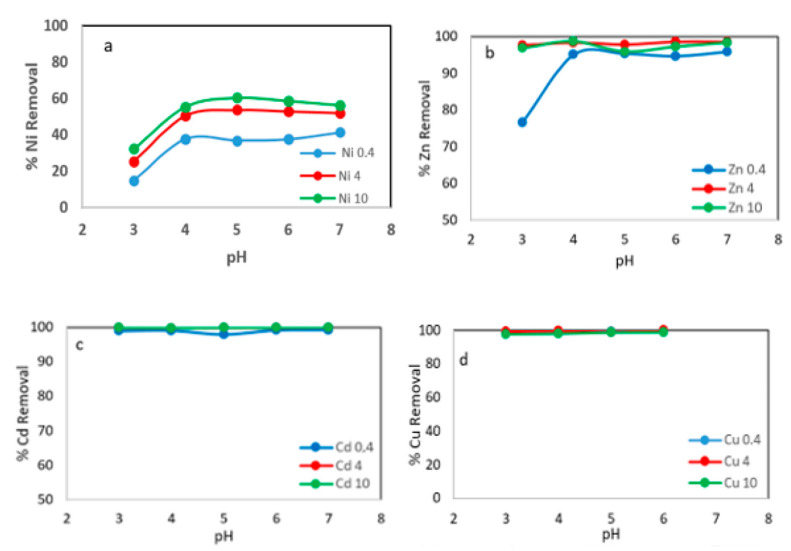
Metals removal onto WS versus initial pH at different adsorbent concentration: 0.4 g L^−1^, 4 g L^−1^ and 10 g L^−1^. (**a**) % Ni removal; (**b**) % Zn removal; (**c**) % Cd removal; (**d**) % Cu removal.

In Figure 4a, it can be seen that the Ni ions removal increased with increasing initial pH; for an adsorbent concentration of 10 g L^−1^ the percentage of metal ions removed was 32.5% and 60.4% for pH 3 and 5, respectively. When smaller amounts of adsorbent were used, the same behavior was observed, although the amount of metal ions removed was lower. Figure 4b shows a removal of 76.6 and 95% of Zn ions for pH 3 and 5, respectively, with an adsorbent concentration of 0.4 mg L^−1^. At higher adsorbent concentrations, the amount of metal ions removed was more than 98%. The adsorption capacity of Cu ions and Cd ions onto WS was not significantly affected by increases in pH in the range of 3 to 7, with removal values in the range of 99 to 100% Figure 4c,d.

The maximum adsorption of metal ions occurred at pH = 5–6. The metal ions adsorption capacity on the WS was not significantly increased with increasing pH above this range, approaching a plateau. The following experiments were conducted at pH 5.5.

A similar behavior was found by Y. Du [31] using two types of mollusk shell powders to remove Pb^2+^, Cd^2+^ and Zn^2+^. Liu et al., 2009 [32], using pulverized bivalve mollusk shells, obtained Cu^2+^ removal efficiencies in the range of 50% to 99.5% at an initial pH between 1 and 5. Ramón de los Santos et al., 2019 [33] used waste oyster shells in the form of biogenic CaCO_3_ nanostructures as adsorbent of Cu^2+^ and Cd^2+^ metal in an aqueous medium; the maximum adsorption capacity obtained was more than 18.6 mg g^−1^ for Cd and 22.7 mg g^−1^ for Cu^2+^ at pH 5. 

The solution’s pH was measured before and after the treatment. When WS particles were added to an acidic aqueous solution, they dissolved the neutralizing acids and increased the dissolved calcium concentration. It was found that a final pH value of 7.7–7.8 was obtained for all the tests carried out with an adsorbent concentration of 0.4 g L^−1^ independent of the initial pH value. The same effect was obtained for higher adsorbent concentrations, with the final pH value being slightly higher. This demonstrates the buffering effect of the WS.

#### 3.3.2. Effect of Contact Time and Dosage

**Figure 5:**
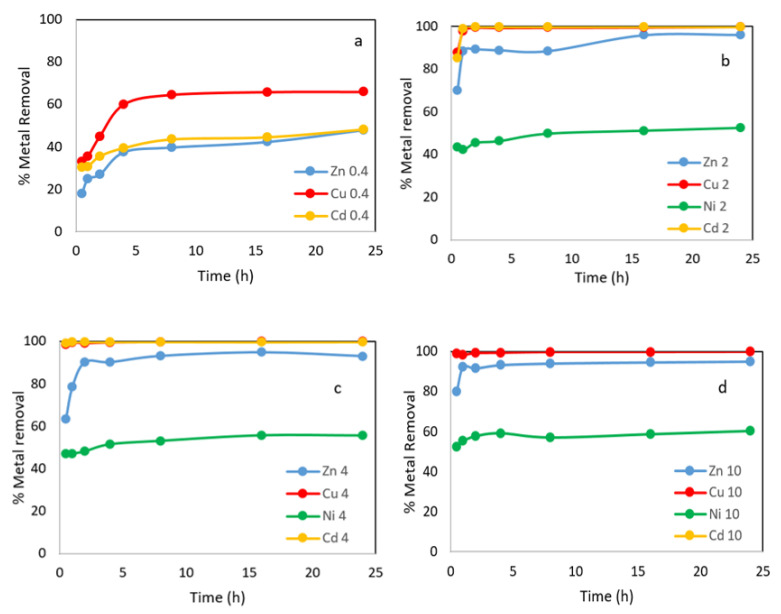
Metals removal onto WS versus time at different adsorbent concentration: 0.4 g L^−1^, 2 g L^−1^, 4 g L^−1^ and 10 g L^−1^. (**a**) % metal removal at 0.4 g L^−1^; (**b**) % metal removal at 2g L^−1^; (**c**) % metal removal at 4g L^−1^; (**d**) % metal removal at 10 g L^−1^.

The tests for Zn^2+^, Cd^2+^ and Cu^2+^ solutions were carried out under the following experimental conditions: initial concentration of 500 mg L^−1^; adsorbent concentration of 0.4–10 g L^−1^; and contact time of 0.08–24 h. In the case of Ni^2+^, the initial concentration was 100 mg L^−1^; the concentration of the WS was 2–10 g L^−1^; and the same range of time (Figure 5).

The results of the batch adsorption experiments showed that the adsorption efficiency of metal ions onto the WS adsorbent increased with increasing time, when the adsorbent concentration was the lowest of those used in this work, 0.4 g L^−1^. A rapid initial absorption of the metallic ions from the water was observed, especially in the first 30 min, during which more than 30% Cd^2+^, 33% Cu^2+^ and 18% Zn^2+^ were removed, and after 8 h more than 46% Cd^2+^, 65% Cu^2+^ and 40% Zn^2+^ were removed; longer times resulted in slight improvements in performance (Figure 5a). The explanation for this behavior is that all the sites on the surface of the sorbent are initially vacant, but with increasing contact time, there is a progressive increase in the bonds between the active sites and the heavy metals, and the metal uptake process become less efficient. 

As the adsorbent concentration increases, the initial uptake is greater, and less time is needed to reach equilibrium. In the case of Cu and Cd, the percentages of metal ions removed were around 99% and slightly more for a concentration of 4 g L^−1^ and 10 g L^−1^, respectively (Figure 5c,d).

Du et al., 2011 [31] tested two types of mollusk shells powders showing different mineralogy, aragonite (clam shells) and calcite (oyster shells), and found that for Cd^2+^, the highest sorption took place in the first 24 h and reached partial sorption equilibrium at 48 h, while the partial sorption equilibrium for Zn^2+^ was reached at 96 h. Zn^2+^ showed a very similar behavior for the two shell types. However, oyster shells removed much less Cd^2+^ than razor clam shells. Núñez et al., 2019 [24] using hydroxyapatite synthesized by wet chemical precipitation as adsorbent, with clam shell waste as raw material, found for Cu^2+^ and Cd^2+^ sorption efficiencies of 65.8% and 81.3%, respectively, in the first 10 min of contact and continued to increase to 80.9% and 92.0% at 1h of contact time. The highest efficiencies that they obtained after 24 h reached 93% for Cu^2+^ and Cd^2+^.

A gradual increase in the percentage of metal removal was observed as the adsorbent dose increased from 0.4 to 10 g L^−1^; thus, the metal uptake increased from 18% to 95% for Zn^2+^, from 30.4% to 99.9% for Cd^2+^ and from 33% to 99.9% for Cu^2+^. However, when the adsorbent concentration increased from 2 to 10 mg L^−1^, the adsorption increased from 52.5% to 60.3% for Ni^2+^. The improvement in adsorption with increasing dosage can be attributed to an increase in surface area and the availability of more binding sites for adsorption.

#### 3.3.3. Effect of Initial Concentration

The effect of the initial concentration on metal ions uptake was investigated by varying the initial concentration of Zn^2+^, Cd^2+^ and Cu^2+^ (100–1000 mg L^−1^) and Ni^2+^ (20–400 mg L^−1^) and different adsorbent concentration of (0.4–10 g L^−1^). All tests were carried out with 24 h of contact time.

**Figure 6:**
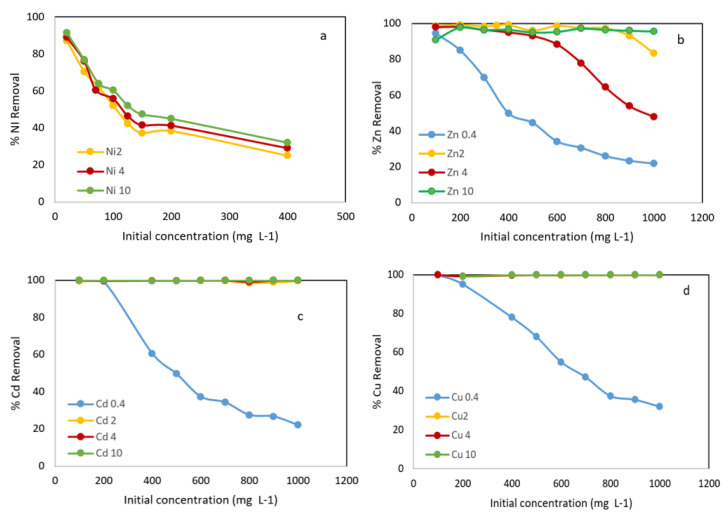
Metals removal onto WS versus initial concentration at different adsorbent concentration: 0.4 g L^−1^, 2 g L^−1^, 4 g L^−1^ and 10 g L^−1^. (**a**) % Ni removal; (**b**) % Zn removal; (**c**) % Cd removal; (**d**) % Cu removal.

As can be seen in Figure 6a, the removal efficiency of Ni^2+^ decreased progressively with increasing initial concentration. When the initial Ni concentration increased from 20 to 400 mg L^−1^, the adsorption decreased from 91.5% to 32.1% using an adsorbent concentration of 10 g L^−1^. In the tests carried out with solutions of other metal ions with initial concentrations (100–1000 mg L^−1^) and using an adsorbent concentration of 0.4 g L^−1^, the variation in the removal efficiency obtained were 99.6–22%, 99–32% and 94.4–21.8% for Cd^2+^, Cu^2+^ and Zn^2+^, respectively (Figure 6b–d). This can be explained by the fact that a given amount of adsorbent has a number of active groups that are able to remove metal ions. As the initial concentration increases, these ions compete among themselves, and there are not enough active groups on the adsorbent surface; therefore, the percentage of metal removal decreases.

The Metal adsorption increased with increasing adsorbent dosage; almost 100% of Cd^2+^ and Cu^2+^ were removed. 

Xu et al., 2019 [20] also found that as the initial Cu^2+^, Cd^2+^ and Pb^2+^ concentration increased, the amount of heavy metal ions adsorbed by the oyster shell increased and the percentage removal decreased. Zhong et al., 2021 [34] using initial concentrations of 100–1100 mg L^−1^ of Pb^2+^ and oyster shell powder as adsorbent observed an increase in adsorption capacity with increasing initial concentration from just over 100 mg g^−1^ until 639.9 mg g^−1^ in the solution initially containing 1100 mg L^−1^ of Pb^2+^.

#### 3.3.4. Characteristics of WS after Treatment with Heavy Metal Solutions

**Figure 7:**
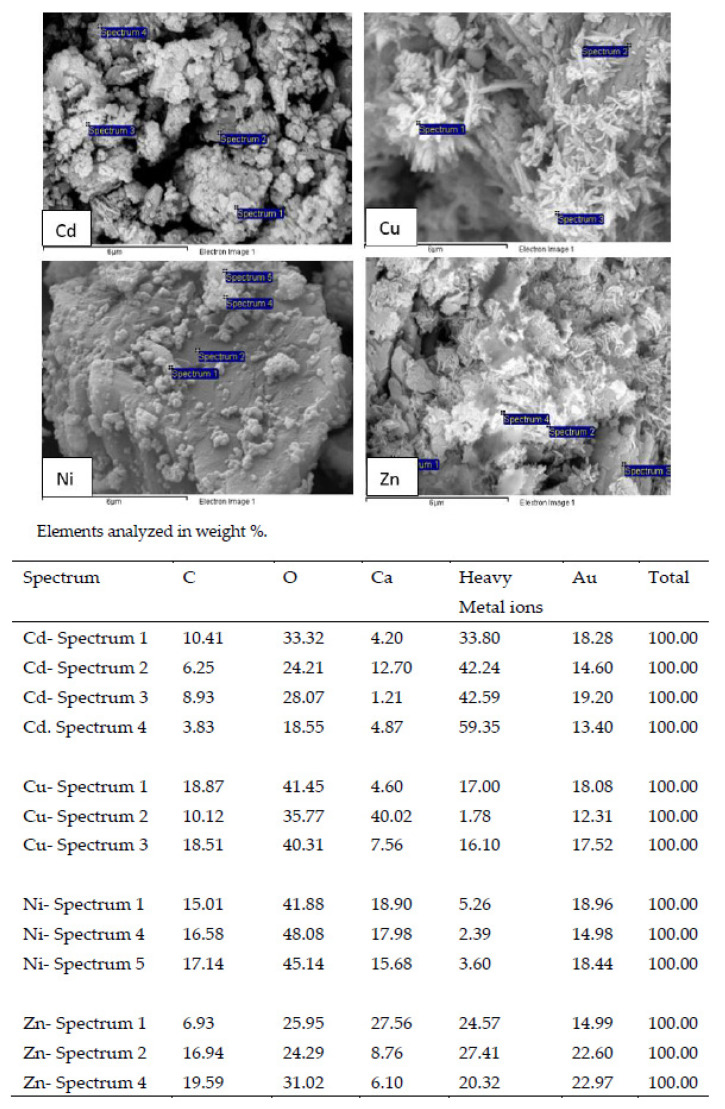
Scanning electron micrograph and EDX analysis of WS after the treatment of the leachate using an adsorbent concentration of 0.4 g L^−1^.

The SEM images of WS after treatment with the metal solution showed the appearance of secondary solids on their surface, indicating that surface precipitation happened during sorption (Figure 7). In the case of the experiments performed with Cd^2+^, according to other authors, it is generally accepted that the sorption by calcium carbonate solid is via surface precipitation of rhombohedral crystals, nearly pure otavite CdCO_3_ [27]. The SEM images of WS treated with Zn^2+^ solutions showed flake-shaped crystallites in aggregates on the sorbent surface, which according to other authors would be hydrozincite crystals [34]. 

The EDAX results showed the high Cd^2+^, Cu^2+^, Ni^2+^ and Zn^2+^ content of these precipitates.

**Figure 8:**
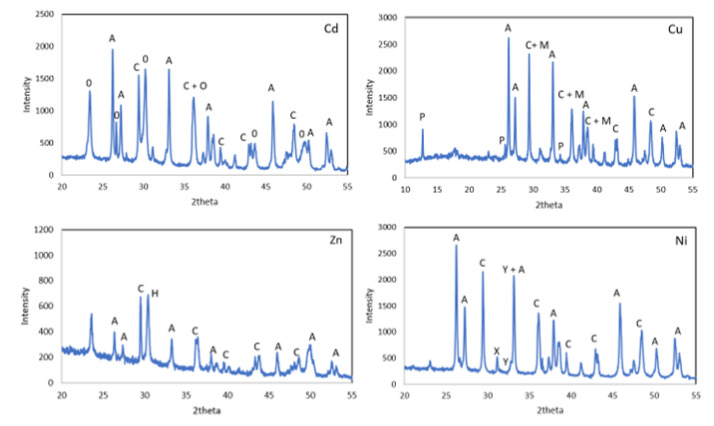
X-ray diffraction patterns of WS after treatment. O—otavite, P—posnjakite, M—malachite, H—hydrozincite, X—Ni_5_(CO_3_)_4_(OH)_2_·4.5H_2_O, Y—Ni_3_(CO_3_)(OH)_4_·4H_2_O, A—aragonite and C—calcite.

The XRD diffraction patterns of WS after treatment with the metal solutions showed the presence of aragonite and calcite in addition to new precipitates such as otavite (CdCO_3_) for Cd and posnjakite and malachite for the tests carried out with Cu solutions. A mixture of basic Ni carbonates with different degrees of hydration Ni_5_(CO_3_)_4_(OH)_2_-4.5H_2_O and Ni_3_(CO_3_)(OH)_4_-4H_2_O, and in the case of the Zn solutions hydrozincite Zn_5_(CO_3_)_2_(OH)_6_ appeared (Figure 8).

#### 3.3.5. Effect of Co-Ions in Solution

Wastewater in general and leachates from different mining and metallurgical facilities may contain different ions that may affect the adsorption of heavy metals onto WS.

A series of binary solutions at pH 5 were prepared by mixing one of the heavy metals ions studied here (Zn, Cd, Cu or Ni) at an initial concentration of 100–400 mg L^−1^ with different metals’ ions (Cu, Zn, Cd or Ni) at concentrations ranging from 100 to 400 mg L^−1^, so that all the solutions prepared contain a total of 500 mg L^−1^. The experiments were performed under the same conditions as in the previous trials. 

The adsorption capacity of WS in a binary system mainly depends on the initial concentration of the primary ion, the co-ion and the initial concentration of co-ions in solution. 

The presence of other metal ions decreased the percentage of Zn^2+^ removal for any of the solutions tested (Table 2). It was observed that a high concentration of Cu^2+^ caused the elimination of only 0.5% of Zn^2+^. The negative effect of co-ions on Zn^2+^ uptake followed the order: Cd^2+^ < Ni^2+^ < Cu^2+^. In the tests carried out with bimetallic solutions with Cd^2+^, it was also observed that the greatest decrease in the removal of this metal took place in the solutions with Cu^2+^ (Table 3). The sequence with the other metals was Zn^2+^ < Ni^2+^ < Cu^2+^. Finally, Cu^2+^ was the metal ion with the highest affinity, so it seemed to be practically unaffected by the presence of the other metal ions; however, a very slight increase in Cu^2+^ yield was observed in the presence of Ni^2+^ (Table 4). Factors that affect the adsorption preference of an adsorbent for metals in a bimetal system are related to the physicochemical properties of the solution such as pH, temperature surface properties of the adsorbent and the properties of the metals such as electronic configuration, electronegativity and ionic radius [35].

Cu^2+^ was the metal ion that most inhibited the removal of Cd^2+^, Zn^2+^ and Ni^2+^. This may be because Cu^2+^ has a 0.73 (Å) radius, similar to Zn^2+^ (0.74 Å) and Ni^2+^ 0.69 (Å) and slightly smaller than Cd^2+^ 0.95 (Å). It has similar electronegativity to Ni^2+^ (1.9 Pauling scale) and slightly higher electronegativity than that of Cd^2+^ (1.69) and Zn^2+^ (1.65). The hydration energy of Cu^2+^ (-2099 kJ mol^−1^) is similar to that of Ni^2+^ (−2096 kJ mol^−1^) and higher than that of Zn^2+^ (−2047 kJ mol^−1^) and Cd^2+^ (−1809 kJ mol^−1^). The covalent index of Cu^2+^ (46) is the same as that of Zn^2+^ (46), slightly lower than that of Ni^2+^ (48) and higher than that of Cd^2+^ (37). All the metals have a valence of 2.

The amount of mmol L^−1^ Ca^2+^ released and the amount of mmol L^−1^ Me^2+^ adsorbed were calculated and compared to see if the molar ratio of Ca^2+^ released to Me^2+^ absorbed was 1:1. In the tests performed, it was found that the amount of Ca2+ released was lower than the amount of metal adsorbed except in some tests performed with Ni, that seemed to indicate that besides the ionic exchange between the Ca^2+^ and Me^2+^ ions, there was another mechanism of adsorption of Me^2+^.

Zhang et al., 2018 [36] found that the adsorption of Cd^2+^ was significantly inhibited in the presence of Cu^2+^, probably due to the competition of this metal ion for the available adsorption sites. They reported that at high initial concentrations (1600 and 3200 mg L^−1^), the amount of Cd^2+^ adsorbed was significantly higher than the amount of Ca^2+^ released, which was likely because the high surface area and negative charge of the CaCO_3_ microparticles allowed for the adsorption of Cd^2+^ on the surface in parallel with the surface dissolution of Ca^2+^ and subsequent CdCO_3_ precipitation. They also found that in tests conducted with elevated Cu^2+^ concentrations, the amount of Cu^2+^ adsorbed was much higher than the amount of Ca^2+^ released and the precipitation of (Cu_2_NO_3_(OH)_3_) took place. Sdiri and Higashi, 2013 [37] studied the simultaneous removal of heavy metals using a natural limestone and found that copper had a strong inhibitory effect over Cd^2+^ and Zn^2+^, which was expected, as copper ions presented a higher affinity on cadmium and zinc ions due to its higher relative binding strength and lower ionization potential. Du et al., 2012 [34] reported that coexisting metals ions in the solution showed a competition effect for Cd^2+^ sorption on a commercial nanoscale aragonite adsorbent. Cu^2+^ showed the most significant effect on Cd^2+^ removal. Köhler et al., 2007 [27] investigated the effect of Zn, Co, Pb, Mg and Ca ions on the uptake of Cd^2+^ by biogenic aragonite. They found different behaviors depending on the type of ion present in the solution; the presence of Pb^2+^ and Zn^2+^ decreased the Cd^2+^ uptake rates, but Ca^2+^ and Co^2+^ did not affect the removal, while Mg^2+^ had a slight enhancing effect. They also observed that the amount of Ca^2+^ released was greater than the quantity of metal ions adsorbed.

### 3.4. Adsorption Isotherms

The tests for Zn^2+^, Cd^2+^ and Cu^2+^ solutions were carried out under the following experimental conditions: initial concentration of 200–1000 mg L^−1^, adsorbent concentration of 0.4 g L^−1^, and contact time of 24 h. In the case of Ni, initial concentration of 20–400 mg L^−1^, concentration of the WS of 2 g L^−1^ and the same range of time.

In order to describe the metal adsorption behavior onto WS, the isotherm data were fitted to the Langmuir and Freundlich adsorption models.

The Langmuir adsorption isotherm is applied to equilibrium adsorption assuming a monolayer adsorption onto a surface with a finite number of identical sites. The Langmuir isotherm is represented by the following equation:

(4) $$\frac{C_{e}}{q_{e}}\;=\;\frac{1}{b\;a_{\max}}\;+\;\frac{C_{e}}{a_{\max}}$$

where *C_e_* is the equilibrium concentration of the metal ion in solution (mg L^−1^), *q_e_* is the amount of metal adsorbed at equilibrium (mg g^−1^) and *b* and *a*_max_ are the Langmuir constants related to the binding constant and the maximum adsorption capacity, respectively. The values were estimated from the intercept and slope of the regression line for different initial metal concentrations.

The essential feature of the Langmuir isotherm can be expressed in terms of the dimensionless separation parameter, *R_L_*. This parameter is indicative of the isotherm shape, which predicts whether an adsorption system is favorable or unfavorable. *R_L_* is defined as:

(5) $$R_{L}=\frac{1}{n\;\left(1+b\;C_{o}\right)}$$

where *b* is the Langmuir constant and *C_o_* is the initial concentration. The *R_L_* value indicates the shape of the isotherm as follows: unfavorable (*R_L_* > 1); linear; favorable (0 < *R_L_* < 1); or irreversible (*R_L_* = 0).

The adsorption data were also tested using the Freundlich isotherm equation:

(6) $$logq_{e}=logK+\frac{1}{n}logC_{e}$$

where *q_e_* is the amount of metal adsorbed at equilibrium (mg g^−1^), *Ce* is the equilibrium concentration of the metal ion in solution (mg L^−1^), *K* is the equilibrium constant indicative of adsorption capacity and *n* is the adsorption equilibrium constant. If the value 1/*n* is below unity, this implies that the sorption process is chemical; if the value is above unity, the sorption is a favorable physical process.

The adsorption parameter values are given in Table 5. The Langmuir isotherm provided the best fit to the experimental data for Cu^2+^, Cd^2+^ and Zn^2+^ with high correlation coefficients (R^2^ > 0.9951); however the data for Ni^2+^ gave a slightly better fit to the Freundlich isotherm (R^2^ = 0.9736). The data showed that the maximum adsorption capacity for Zn^2+^, Cd^2+^ and Cu^2+^, a_max_, was 526.32 mg g^−1^, 555.56 mg g^−1^ and 769.23 mg g^−1^, respectively.

The lower adsorption capacity of Ni^2+^ may be due to the fact that the precipitation of Ni_5_(CO_3_)4(OH)^2−^4.5H_2_O and Ni_3_(CO_3_)(OH)^4−^ 4H_2_O is less favored than the precipitation of otavite for Cd, posnjakite and malachite for the tests performed with Cu solutions, and hydrozincite in the case of Zn solutions.

Xu et al., 2019 [20] studied the removal of Cu^2+^ and Cd^2+^ by oyster shells and also found that Cd^2+^ and Cu^2+^ best fit the Langmuir isotherm model. Wu et al., 2014 [38] investigated the removal of Cu^2+^ by oyster shell powder, in particular, the adsorption behavior differences between the prismatic (PP) and nacreous (NP) shell layers. The adsorption of Cu^2+^ to the NP layer correlated better with a Langmuir isotherm for the initial concentration range (5–200 mg L^−1^). However, they found a different behavior for the PP layer; when the study was carried out with low initial concentrations (5–30 mg L^−1^), there was a better fit for the Langmuir model and when the range of concentrations was greater (30–200 mg L^−1^), they found a strong agreement with a heterogeneous Freundlich model. However, Núñez et al., 2019 [24] found that the removal of Cd^2+^ and Cu^2+^ using hydroxyapatite synthesized by wet chemical precipitation using clam shell waste as feedstock agreed well with both models.

Ahmad et al., 2012 [39] studied the removal of Cu^2+^ and Cd^2+^ by other adsorbents composed mainly of calcium carbonate such as eggshell or coral. They reported that the maximum amounts of Cu^2+^ and Cd^2+^ adsorbed were 32.3 and 4.47 mmol kg^−1^ for eggshell and 6.77, 5 and 1.03 mmol kg^−1^ for coral wastes, respectively. 

The R_L_ values for adsorption on waste shell at the lowest concentrations were 0.0731, 0.0042, 0.0682 and 0.7013 for Cd, Cu, Zn and Ni ions, respectively, while for the highest concentration studied, the values varied between 0.0073 and 0.1050. The data thus obtained represent a favorable adsorption.

The standard Gibbs free energy changes (∆Go) for the adsorption process can be calculated using the following equation:

∆Go = − RT Ln b(7)

where b is the Langmuir constant, R is the gas constant and T is temperature. The negative free energy values indicate that the process is both viable and spontaneous.

### 3.5. Kinetics Adsorption Studies

Several models can be used to express the mechanism of solute sorption onto a sorbent. The pseudo-second-order rate expression was used to describe chemisorption involving valency forces through the sharing or exchange of electrons between the adsorbent and adsorbate as covalent forces, and ion exchange. Although there are many factors which influence the sorption capacity, including the initial sorbate concentration, the reaction temperature, the solution pH value, the sorbent particle size and dose and the nature of the solute, a kinetic model is concerned only with the effect of observable parameters on the overall rate. 

The pseudo-second-order kinetic model can be described by the following equation:

(8) $$\frac{t}{Q_{t}}=\left[\frac{1}{K_{2}{\;Q}_{e}^{2}}\right]+\left(\frac{1}{Q_{e}}\right)t$$

where Q_t_ is the amount (mg g^−1^) of material adsorbed at time t, Q_e_ is the adsorption capacity (mg g^−1^) and k_2_ is the rate constant (g mg^−1^ h^−1^) of the pseudo-second-order model. From the slope and intercept of the straight line obtained by plotting t/Q_t_ versus time, the value k_2_ and the equilibrium capacity (Q_e_) were determined. The initial sorption rate, in the pseudo-second-order model, as h = Q_t_/t when t approaches 0, h (mg gL h^−1^), is h = K_2_ Q_e_^2^.

The rate kinetics of metal ion adsorption onto WS at the initial metal ion concentration of 100 mg L^−1^ for Ni^2+^ and 500 mg L^−1^ for Cd^2+^, Cu^2+^ and Zn^2+^ and different adsorbent concentration were analyzed using pseudo-second-order models.

The results showed that the adsorption data could fit the pseudo-second-order model for most of the cases studied since it presented a very high linearity, R^2^ > 0.99, except for Ni^2+^ and Cd^2+^ when 2 g L^−1^ of adsorbent concentration was used (Table 6). The equilibrium capacity (Q_e_) of Cd^2+^, Cu^2+^ and Zn^2+^ and Ni^2+^ onto WS, followed the order Cd^2+^ = Cu^2+^ > Zn^2+^ > Ni^2+^ for high adsorbent concentrations; however, when lower concentrations were used, the following orders Cu^2+^ > Zn^2+^ > Cd^2+^ > Ni^2+^ and Cu^2+^ > Zn^2+^ > Cd^2+^ were found for 2 g L^−1^ and 0.4 g L^−1^, respectively, similar to those obtained from the Langmuir isotherm.

Sdiri et al., 2012 [14] calculated and measured the amounts of sorbed solute at equilibrium and suggested that the removal process of Cd, Cu and Zn ions by different natural limestones fitted the pseudo-second-order kinetic model. Hsu 2009 [40] studied the removal kinetics of Cu^2+^ and Ni^2+^ by pulverized oyster shells at different temperatures. He found that it fitted well to the pseudo-second-order model, with the initial maximum sorption rates (h) for Cu^2+^ and Ni^2+^ being 3.896 mg g^−1^ min^−1^ (60 °C) and 6.219 mg g^−1^ min^−1^ (60 °C), respectively. Hassan et al., 2020 [41] also studied the adsorption of Co, Zn, Pb and Hg ions on eggshell surfaces and found that the adsorption obeyed second-order kinetics.

### 3.6. Treatment of Mining and Metallurgical Leachates

Experiments were carried out at different reaction times, 8 and 24 h, using adsorbent concentrations of 0.4 g L^−1^ and 0.2 g L^−1^ under the same conditions as in the previous trials. 

In the tests carried out with synthetic solutions with 100 mg L^−1^ of a single metal, the removal efficiency after 24 h of treatment was 99.9%, 99.5% and 94.4% for Cu, Cd and Zn ions, respectively. However, in the tests with the bimetallic solutions and the same metal ions concentration, it was observed that Cu^2+^ was practically unaffected, with only a 2% decrease in efficiency in the presence of Cd^2+^.

In the treatments with mining and metallurgical leachates, different behaviors were observed depending on the type of leachate; the Cu^2+^ removal efficiency for the S leachate was around 97% in the three tests performed and from 43% to 57% for the O leachate with an adsorbent concentration of 0.4 g L^−1^ (Table 7). Sample P, which originally contained the highest amount of Cd^2+^ and practically no Cu^2+^, obtained the best performance in Cd^2+^ removal. In general, removal efficiencies were much lower than those found with the synthetic solutions, suggesting that they are not only affected by the presence of these ions but that in real wastewater, there are anions, cations and organic matter that can affect the removal of the metal ions studied in this work.

Leachate O had an arsenic ions content of 111.85 μg L^−1^; after the three treatments it had a maximum content of 4 μg L^−1^. The World Health Organization (WHO) Guidelines for Drinking-Water Quality recommend limits of 10 μg L^−1^. The P and S leachates had very high arsenic ions concentrations, 6.71 mg L^−1^ and 5.72 mg L^−1^, respectively. After treatment with WS, it was observed that the removal efficiency was important, varying between 62.8% and 89.49%; however, they still had a high content of these ions that could be improved by increasing the adsorbent concentration, since only 0.4 g L^−1^ was used in the best of cases.

Wang and Zhu 2019 [42] studied the removal of As(V) ions from aqueous solutions using CaO, CaF_2_ and CaCO_3_; they found that the main mechanism for the removal of As(V) ions by calcium-bearing materials was the formation of insoluble calcium arsenate salt generated by arsenate anions and calcium ions. Ayala and Fernandez 2020 [43] studied the capacity of four industrial waste materials originating from steelmaking processes (slags) and from gas treatment at a thermal power plant (fly ash and gypsum) to remove As ions from a leachate from the spoil heap of an abandoned mercury mine. The mechanism that they proposed, when using residues with high calcium contents, was the precipitation of the Ca–As compounds due to the solubilization of Ca ions that leads to an increase in pH.

Se ions contents in leachates were lower than 66.6 μg L^−1^ and it was found that the efficiency of WS did not reach 20%. The concentration of selenium ions in natural water is usually below 3 μg L^−1^, the lowest permissible limit suggested by WHO being 10 μg L^−1^. So, to reach these values, a greater amount of adsorbent would be needed.

The Se ions removal mechanism is similar to that of As ions since it can form Ca–Se compounds [44].

The amount of Ca^2+^ released was higher than the quantity of heavy metals ions adsorbed onto WS, which confirmed the data obtained in the treatment of bimetallic solutions. This release of calcium ions increased the final pH value by one unit or slightly more. The amount of Na, K and Mg ions released came mainly from the metallic chlorides adhered to the WS, since they were not washed prior to their use as adsorbents (Table 8).

Several authors studied the desorption of these metals retained on mollusk shells and eggshells using different leaching agents. They found that the adsorption process was usually irreversible because the adsorption mechanism was surface precipitation. Therefore, the leaching of metal from the metal-laden adsorbent to the environment seems negligible and could be considered a nonhazardous waste [45,46]. However, in a future study, the leaching test will be carried out under more drastic conditions in order to evaluate the possible release of these metals, in which case the encapsulation of metal-loaded WS by solidification/stabilization techniques would be proposed.

## 4. Conclusions

The results of this study show that the calcium carbonate from WS is an effective and low-cost adsorbent for the removal of heavy metals ions in aqueous solution. The percentage of metal ions removal gradually increases with increasing concentration and the maximum adsorption of metal ions occurred at pH = 5–6.

The presence of co-ions suppressed the uptake of heavy metals ions; Cu^2+^ was the metal that most inhibited the removal of Cd^2+^, Zn^2+^ and Ni^2+^. The experimental data for Cu^2+^, Cd^2+^ and Zn^2+^ best fitted the Langmuir isotherm model, while Ni^2+^ best fitted the Freundlich isotherm model. The affinity of WS for Ni^2+^ was always lower than that for Cu, Cd and Zn ions for both single and bimetal solutions.

The present study demonstrates that it is possible to carry out an efficient and economic treatment of mining and metallurgical leachates by simultaneously removing several heavy metals’ ions such as Cu, Ni, Zn, Cd, Ni, As and Se using WS as adsorbent.

## Figures and Tables

**Table 1:** Metal ions concentrations analyzed by ICP of three mining and metallurgical leachates.

	Major Component			Minor Component			
	(mg L^−1^)			(μg L^−1^)			
	O	P	S		O	P	S
Cu	27.66	0.02	8.16	Mn	2.91	25.82	2.98
Ni	8.25	20.82	13.23	Se	43.39	66.64	44.28
Zn	22.29	16.27	15.11	Hg		0.46	
Cd	28.2	67.61	16.69	Ag		12.9	
As	0.11	6.71	5.72	Pb	4.24		
Na	15.42	15.54	14.71	U	4.4		
Mg	74.04	1.64	15.55	Fe	4.65	2.6	3.1
K	6.58	22.56	2.13	Sb	5.87	31.16	4.55
Ca	117.3	42.89	86.98	B	20.37	205.17	26.55
				Al		407.87	
				Mo		5.67	
				Sr	362.84	190.36	361.4
				Ba	55.73	8.11	12.61

**Table 2:** Effect of co-ions on the Zn^2+^ removal.

Metal	Me0	Zn0	% Me	% Zn	∑(mM)	mM Me	mM Zn	∑ (mM)	Ca (mM)	Difference	pH_final_
	(mM)	(mM)	Remov.	Remov.		Remov.	Remov.	Remov.	Released	mM	
Zn only		1.53		94.4			1.44				7.89
Cd	3.56	1.53	80.4	86.3	5.09	2.86	1.32	4.18	2.19	1.99	7.10
Cu	6.29	1.53	94.5	0.5	7.82	5.94	0.01	5.94	3.07	2.88	6.07
Ni	6.82	1.53	6.25	21.0	8.35	0.43	0.32	0.75	0.85	−0.10	7.44
Zn only		3.06		85.1			2.60				7.64
Cd	2.67	3.06	80.4	85.8	5.73	2.39	1.44	3.83	2.42	1.40	7.14
Cu	4.72	3.06	92.3	6.0	7.78	4.35	0.18	4.54	3.05	1.49	6.72
Ni	5.11	3.06	9.0	39.0	8.17	0.46	1.20	1.65	2.02	−0.37	7.46
Zn only		4.59		69.7			3.20				7.44
Cd	1.78	4.59	89.0	51.7	6.37	1.58	2.37	3.95	2.55	1.40	7.17
Cu	3.15	4.59	99.0	13.3	7.74	3.11	0.61	3.73	2.41	1.31	6.95
Ni	3.41	4.59	14.25	48.3	8.00	0.48	2.22	2.70	2.71	−0.01	7.47
Zn only		6.12		49.9			3.05				7.08
Cd	0.89	6.12	81.5	39.8	7.01	0.72	2.43	3.15	2.66	0.49	7.14
Cu	1.57	6.12	99.7	20.6	7.69	1.57	1.26	2.83	2.81	0.02	7.27
Ni	1.7	6.12	18.7	47.5	7.82	0.32	2.90	3.22	3.20	0.023	7.33
Zn only		7.64		44.5			3.40				7.05

**Table 3:** Effect of co-ions on the Cd^2+^ removal.

Metal	Me0	Cd0	% Me	% Cd	∑(mM)	mM Me	mM Cd	∑ (mM)	Ca (mM)	Difference	pH_final_
	(mM)	(mM)	Remov.	Remov.		Remov.	Remov.	Remov.	Released	mM	
Cd only		0.89		99.5			0.88				7.90
Cu	6.29	0.89	72.8	1.0	7.18	4.58	0.01	4.59	3.00	1.59	6.08
Zn	6.12	0.89	39.8	81.5	7.01	2.43	0.72	3.15	2.66	0.49	7.14
Ni	6.82	0.89	4.5	85.4	7.71	0.31	0.76	1.07	0.99	0.079	7.55
Cd only		1.78		99.4			1.77				7.76
Cu	4.72	1.78	90.2	1.8	6.50	4.35	0.03	4.38	2.94	1.44	6.69
Zn	4.59	1.78	51.7	89.0	6.37	2.37	1.58	3.95	2.55	1.40	7.17
Ni	5.11	1.78	7.7	76.2	6.89	0.39	1.35	1.75	2.02	−0.28	7.69
Cd only		2.67		89.7			2.39				7.18
Cu	3.15	2.67	95.8	6.3	5.82	3.04	0.17	3.21	2.16	1.05	6.82
Zn	3.06	2.67	80.8	89.5	5.73	1.44	2.39	3.82	2.42	1.40	7.14
Ni	3.41	2.67	8.3	62.5	6.08	0.28	1.67	1.95	1.65	0.30	7.21
Cd only		3.56		80.6			2.87				6.89
Cu	1.57	3.56	97.6	13.3	5.13	1.53	0.46	2.00	1.62	0.37	7.36
Zn	1.53	3.56	86.3	80.4	5.09	1.32	2.86	4.18	2.19	2.00	7.10
Ni	1.7	3.56	3.8	50.4	5.26	0.06	1.79	1.86	1.79	0.07	7.26
Cd only		4.45		49.7			2.21				6.66

**Table 4:** Effect of co-ions on the Cu^2+^ removal.

Metal	Me_0_	Cu0	% Me	% Cu	∑(mM)	mM Me	mM Cu	∑ (mM)	Ca (mM)	Difference	pH_final_
	(mM)	(mM)	Remov.	Remov.		Remov.	Remov.	Remov.	Released	mM	
Cu only		1.57		99.9			1.57				6.55
Cd	3.56	1.57	13.0	97.6	5.13	0.46	1.53	1.20	1.62	0.37	7.36
Zn	6.12	1.57	20.6	99.7	7.69	1.26	1.57	2.83	2.81	0.02	7.27
Ni	6.82	1.57	3.8	99.7	8.39	0.25	1.57	1.82	1.62	0.20	7.45
Cu only		3.15		97.1			3.06				6.47
Cd	2.67	3.15	6.3	96.8	5.82	0.17	3.04	3.21	2.16	1.05	6.82
Zn	4.59	3.15	13.3	96.0	7.74	0.61	3.11	3.72	2.41	1.31	6.95
Ni	5.11	3.15	4.2	97.6	8.26	0.21	3.07	3.28	2.17	1.11	6.66
Cu only		4.72		92.5			4.37				6.02
Cd	1.78	4.72	1.8	92.2	6.50	0.03	4.35	4.38	2.94	1.44	6.69
Zn	3.06	4.72	6	92.3	7.78	0.18	4.36	4.54	3.05	1.49	6.72
Ni	3.41	4.72	6.3	92.9	8.13	0.14	4.39	4.52	2.94	1.58	6.36
Cu only		6.29		82.4			5.18				5.77
Cd	0.89	6.29	1.0	72.8	7.18	0.09	4.58	4.59	3.00	1.59	6.08
Zn	1.53	6.29	0.5	82.1	7.82	0.01	5.16	5.17	3.06	2.11	6.07
Ni	1.7	6.29	4.5	80.3	7.99	0.08	5.06	5.13	3.32	1.81	6.03
Cu only		7.87		70.0			5.51				5.66

**Table 5:** Langmuir and Freundlich adsorption isotherm constants.

Metal	*a*_max_ (mg g^−1^)	*b* (L mg^−1^)	R^2^	∆G (KJ mol^−1^)	*K*	1/*n*	R^2^
Ni	54.345	0.0213	0.9337	−17,349	6.453	2.830	0.9736
Zn	526.32	0.1367	0.9984	−27,350	226	7.032	0.7782
Cd	555.56	0.1268	0.9951	−23,301	360	12.469	0.6521
Cu	769.23	1.1818	0.9966	−22,165	389	8.137	0.6572

**Table 6:** Kinetic parameters.

	Pseudo-Second-Order				
Adsorbent Concentration (g L^−1^)	Metal	R^2^	K_2_	Q_e_	h
			(g mg^−1^ h^−1^)	(mg g^−1^)	(mg g^−1^ h^−1^)
	Zn	1	6.176	47.62	588.23
10	Cu	1	33.333	50	3333.33
	Cd	1	333.333	50	33,333.33
	Ni	0.9996	3.396	6.01	40.8
	Zn	0.9998	3.269	117.65	769.23
4	Cu	1	40	125	10,000
	Cd	1	400	125	100,000
	Ni	0.9998	1.874	14.04	52.63
	Zn	0.9993	1.864	243.9	909.09
2	Cu	1	20	250	10,000
	Cd	0.9727	0.61	163.93	200
	Ni	0.8388	0.8388	4.93	0.04
	Zn	0.9943	0.364	625	454.54
0.4	Cu	0.9994	0.75	833.33	1250
	Cd	0.9977	0.667	625	833.33

**Table 7:** % Metal ions removal and final pH of the leachate treatment with WS.

	O 8 h/0.4	O 24 h/0.4	O 24 h/0.2	P 8 h/0.4	P 24 h/0.4	P 24 h/0.2	S 8 h/0.4	S 24 h/0.4	S 24 h/0.2
Ni	8.09	6.64	5.47	16.56	16.81	17.32	10.79	15.25	16.57
Cu	43.55	56.71	45.98	95.01	97.9	95.36	97.56	98.51	96.86
Zn	9.3	7.46	6.75	30.93	41.71	30.53	20.71	30.61	28.35
Cd	11.39	10.78	9.08	32.06	40.23	31.59	22.2	32.38	28.53
As	96.14	97.82	96.28	62.8	89.49	66.86	65.61	86.13	70.69
Se	0.72	8.14	2.47	13.46	15.47	11.01	16.18	20.26	18.64
pH_final_	6.37	6.45	6.36	6.57	6.76	6.50	6.59	7.01	6.62

**Table 8:** Release of Na^+^, K^+^, Ca^2+^ and Mg^2+^ ions due to adsorption of Zn^2+^, Cd^2+^, Ni^2+^ and Cu^2+^ onto WS after 24 h of treatment with an adsorbent concentration of 0.4 g L^−1^.

	Metal Bound (mM)				Amount of Cation Released (mM)			
Leachate	Zn^2+^	Cd^2+^	Ni^2+^	Cu^2+^	Na^+^	K^+^	Ca^2+^	Mg^2+^
O	0.00003	0.02705	0.00934	0.24683	0.42211	0.01469	0.35250	0.11422
P	0.10373	0.24197	0.05964	0.00025	0.37579	0.01341	0.29651	0.06168
S	0.07067	0.04807	0.03438	0.12655	0.46773	0.01280	0.94008	0.05000
